# A Case Series of a Novel Approach in the Treatment of Rosacea: Use of Botulinum Toxin and Intense Pulsed Light for the Treatment of Erythematotelangiectatic Rosacea

**DOI:** 10.1111/jocd.16774

**Published:** 2025-01-07

**Authors:** Cesar Gonzalez Ardila, Laura A. Colorado Franco, Manuel Franco, Andrea Galeano, Angie Julieth Holguin Molina, Julio R. Amador, Marco Rocha

**Affiliations:** ^1^ Private Dermatology Clinic Bogotá Colombia; ^2^ Private Aesthetic Medicine Clinic Bogotá Colombia; ^3^ Universidad Del Bosque Bogotá Colombia; ^4^ Federal University of São Paulo Brazil

To the editor,

Rosacea is a chronic inflammatory skin disorder, prevalent in individuals with light skin, most of the times difficult to treat with conventional treatments [1]. Although both Botulinum neurotoxin Type A (BoNT/A) and Intense Pulsed Light (IPL) separately have proven to be safe and effective alternatives, studies have shown that the combination of therapies may lead to better treatment outcomes [2]. In our study, we found an improvement in erythematotelangiectatic rosacea using the combination of BoNT/A and IPL.

Intense Pulsed Light (IPL) is a well‐established light‐based therapy for various skin diseases, particularly in alleviating erythematous telangiectasia, papules, and pustules. IPL operates using a broad spectrum of light, with wavelengths ranging from 500 to 1200 nm modifiable through various filters [3]. Its effects include the following: mast cell membrane stabilization, inhibiting their degranulation, and the release of cytokines and mediators (histamine, tryptase, MMP‐9, and LL‐37), ultimately achieving a therapeutic effect [3]. Despite its effectiveness, it is commonly noted that monotherapy does not achieve long‐lasting results in this condition. In contrast, patients who receive combination treatments, including (BoNT/A), experience improvements that persist for a much longer duration [4].

Botulinum neurotoxin (BoNT) blocks the release of acetylcholine and modulates other neuropeptides, such as vascular endothelial growth factor, substance P, and calcitonin gene‐related peptide, which influences vasodilation and inhibits the release of cathelicidin and inflammatory mediators promoting an anti‐inflammatory effect that alleviates facial redness. Inhibits mast cell degranulation, stimulating an anti‐inflammatory effect [5]. The effect of BoNT/A injection is generally gradual after 3 days reaching efficacy peak 2 weeks after injection. If improvement is not satisfactory at the 2‐week follow‐up, complementary therapy can be administered. To avoid the thermal effect that accelerates the diffusion of local BoNT/A, the skin should be cooled after photoelectric treatment. Alternatively, photoelectric treatment can be administered at least 2 weeks after injection. Patients with severe lesions should be considered systemic medication to quickly control inflammation [6].

Therefore, considering the previously mentioned mechanisms of action, combining these two therapies could result in a potential synergistic effect, likely leading to better outcomes for our patients.

We reviewed the medical records of 14 patients diagnosed with erythematotelangiectatic rosacea, all over 18 years old. As exclusion criteria, they could not have previously received topical therapy, injectables, or IPL treatment for their initial condition.

They received treatment with one session of IPL at 560 nm and 14 joules followed by injections of BoNT/A at 2.5 U per 0.1 mL. The evaluation involved reviewing photographs of the patients taken during the initial consultation and 4 weeks after treatment. Two experienced dermatologists assessed the photographs using the Global Physician Assessment (GPA) and assigned the respective scores. When no agreement was reached on the photographs along with the scores, a consensus was made for the final score. Each patient had signed informed consent for the taking of photographs and publications.

A total of 14 medical records were reviewed, consisting of 13 women and 1 man, with an average age of 43.7 and Fitzpatrick skin type III. All patients had erythematotelangiectatic rosacea. The severity of rosacea was graded from 0 to 3 as follows: 0—normal, 1—mild, 2—moderate, 3—severe. Two patients were classified as severe, six as moderate, five as mild, and one was categorized as normal at baseline. The average score at the beginning was 1.64 (SD 0.84), while the follow‐up score averaged 0.86 (SD 0.66), showing a global improvement with the treatment. Of the 14 patients, most of the patients (64.3%) showed improvement from their initial condition, and three of them reached a status classified as normal. No patients remained in the severe category, which are the ones with worse quality of life; 2 were classified as moderate, 8 as mild, and 4 as normal. Twelve of the 14 patients ended up between mild and normal (Table 1) (Figure 1).

**TABLE 1:** Baseline characteristics and severity of Rosacea before and after 4 weeks of treatment.

Variable			*n* = 14		%
Baseline characteristics					
Average age (SD)			43.7		100
Sex					
Female			13		928 571 429
Male			1		714 285 714
Fitzpatrick			III		100

	Before	After	% difference	IC 95%	*p*
Severity of erythema and telangiectasias					
Improved	0	9	64.29%	39.19 to 89.39	0.0039
Normal or mild	6	12	42.86%	16.93 to 68.78	0.0313
Severe	2	0	14.29%	−32.62 to 4.04	0.5

**FIGURE 1:**
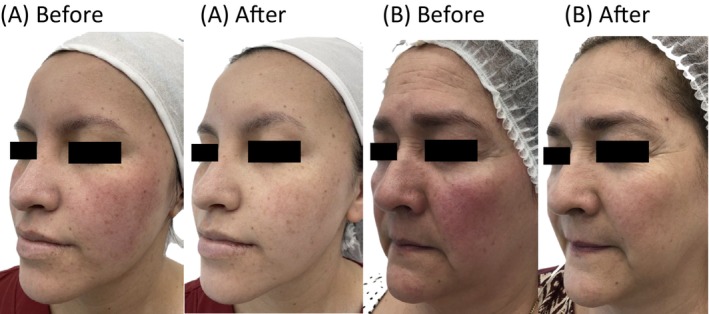
Rosacea. Photographs of women showing before and after treatment with BoNT and IPL. Results at Week 4. In photograph (A), the patient shows significant improvement in erythema and telangiectasias, particularly on the cheeks and nose. In photograph (B), the improvement of erythema and telangiectasias extends to the cheeks, nose, chin, and forehead.

At 4 weeks, a satisfaction survey was conducted with the patients regarding erythema and telangiectasias, 11 of the 14 surveyed patients scored 4 of 5 points or higher.

To our knowledge, we present the first approach to the use of BoNT/A and IPL in the Latin American population with erythematotelangiectatic rosacea with Fitzpatrick skin type III, opening up a range of therapeutic options that could be useful and available for our patients. However, the limitations of this study included the small sample size, lack of a control group or comparator, and short duration of follow‐up (4 weeks), which limits insights into the long‐term efficacy and safety of the combination therapy. For the future, we recommend controlled trials with larger sample sizes and longer follow‐up periods to confirm these findings.

## Conflicts of Interest

The authors declare no conflicts of interest.

## Data Availability

The data that supports the findings of this study are available in the supplementary material of this article.
